# Intrapartum Fetal Heart Rate Pattern Evolution Associated With Septuple Nuchal Cord Loops: A Case Report

**DOI:** 10.1155/crog/9925453

**Published:** 2025-10-20

**Authors:** Masanori Sugimoto, Masahiro Nakao, Masafumi Nii, Kohei Sakakibara, Saki Kotaka, Ryo Nimura, Tokihiro Senda, Yoshiki Maeda, Toru Hirata, Tomoaki Ikeda

**Affiliations:** ^1^Department of Obstetrics and Gynecology, Kuwana City Medical Center, Kuwana, Mie, Japan; ^2^Department of Obstetrics and Gynecology, Mie University Graduate School of Medicine, Tsu, Mie, Japan

**Keywords:** fetal heart rate, nuchal cord, prenatal diagnosis, ultrasonography

## Abstract

**Background:**

Although a nuchal cord is a common observation, multiple nuchal cord loops are rare. A few reports suggest that multiple nuchal cords are associated with adverse perinatal outcomes, such as cesarean or operative vaginal deliveries for fetal distress and neonatal asphyxia. However, there are controversies regarding antenatal detection and perinatal management of multiple nuchal cord loops. Herein, we report a case of live vaginal birth with septuple nuchal cords demonstrating a distinct pattern of fetal heart rate evolution during labor.

**Case Presentation:**

A 31-year-old primigravida was referred to our facility at 34 weeks of gestation with multiple nuchal cords. Transabdominal ultrasonography revealed a three-vessel cord with extreme loops around the fetal neck. The antenatal course was uneventful with appropriate fetal growth, amniotic fluid index, and reactive nonstress testing until the last checkup. The patient underwent labor at 38 weeks of gestation. The admission test on the fetal heart rate tracing indicated nonrecurrent but mild variable decelerations with an “atypical” feature. Subsequently, there were recurrent, severe decelerations and an elevation of the baseline rate during the second stage of labor. The patient delivered a male infant, weighing 2455 g (10.5 percentile of normal weight) vaginally via vacuum extraction. The umbilical arterial gas analysis at birth showed a pH of 7.31, a partial pressure of oxygen of 23.4 mmHg, a base excess of −6.4, and 1/5-min Apgar scores of 8/9. The cord was 112 cm long with septuple nuchal cords.

**Conclusions:**

As umbilical cord abnormalities have a risk of fetal heart rate compromise in the prelabor or first stage of labor, prenatal detection of umbilical cord pathology may be beneficial for the early recognition of potential fetal compromise and prompt decision-making for operative intervention.

## 1. Introduction

A nuchal cord (NC) commonly occurs in 19%–24% of deliveries [1]. The incidence of a single loop is 16%, whereas those of double, triple, and quadruple NCs at delivery are reportedly 3%, 1%, and <1%, respectively [1]. Two meta-analyses indicated that umbilical cord entanglement is associated with a significantly higher incidence of fetal distress, umbilical cord blood *pH* < 7.1 at birth, and a neonatal *Apgar* *score* < 7 in the first minute after birth [1, 2]. Furthermore, multiple loops are associated with an increased likelihood of cesarean section and low Apgar scores at 1 and 5 min [1]. Additionally, Schreiber et al. evaluated 42,798 cases with singleton vertex pregnancies who delivered vaginally based on the number of loops and stated that three loops of NC were associated with an increased risk of operative deliveries and low Apgar scores [3].

Deceleration of fetal heart rate (FHR) is a prominent feature of fetal physiological adaptation to hypoxic and mechanical stress during labor [4, 5]. The presence of umbilical cord complications increases the risk of abnormal FHR parameters, including variable and late decelerations [6, 7], which may lead to fetal metabolic acidosis [8]. However, there are controversies regarding the perinatal management of multiple NC loops. In the current practice, the International Society of Ultrasound in Obstetrics and Gynecology guidelines do not mention routine prenatal assessment for the NC [9], nor are early deliveries recommended for any form of umbilical cord complication other than vasa previa [10].

Herein, we present a rare case of live vaginal birth with a prenatal diagnosis of extreme NC and a long cord, demonstrating a distinct pattern of evolution of FHR during labor. Written informed consent was obtained from the patient for the publication of this case report.

## 2. Case Presentation

**Figure 1:**
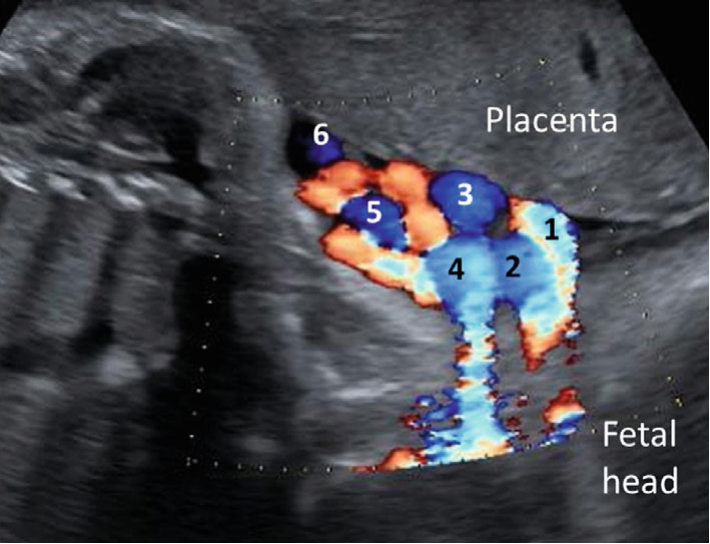
Coronal imaging of the fetal neck with color Doppler at 34 weeks of gestation. Numbers on the color Doppler show sextuple loops of nuchal cord.

A 31-year-old primigravid woman was referred to our hospital at 34 weeks of gestation for perinatal management of NC with multiple loops. The patient had a history of atopy that was well controlled with oral levocetirizine hydrochloride (5 mg once daily), epinastine hydrochloride (20 mg once daily), and as-needed topical dexamethasone. The patient had no relevant family or smoking history and underwent regular antenatal checkups at a primary clinic with no complications during early pregnancy. However, the extreme loops of the NC were observed at 33 weeks of gestation. Subsequently, she was referred to our hospital at 34 weeks of gestation. Color Doppler sonography performed at her first visit revealed a three-vessel cord with six loops of entanglement around the fetal neck (Figure 1). The estimated fetal weight was 2029 g (27.4 percentile), with an amniotic fluid index (AFI) of 8 cm. The patient revisited the hospital with the chief complaint of decreased fetal movement at 35 weeks of gestation; however, no evidence of a non-reassuring status was observed with a normal modified biophysical profile. Subsequent fetal growth was maintained within a normal range, at 42.1 percentile at 38 0/7 weeks of gestation, but with a decrease of AFI to borderline (**AFI = 5.2** cm).

**Figure 2:**
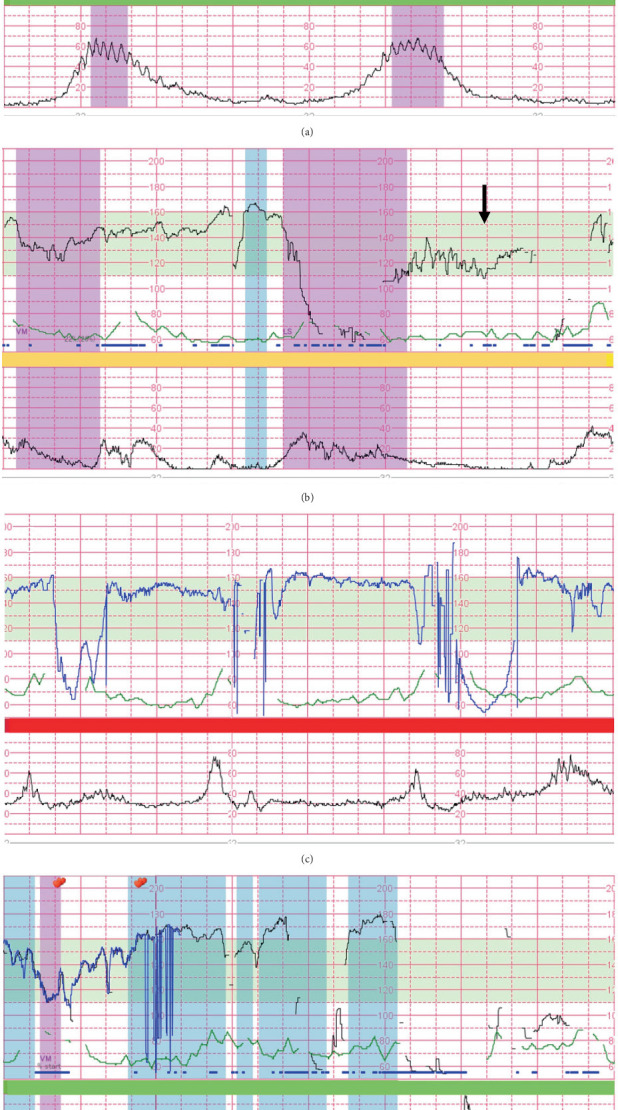
Fetal heart rate tracings during labor. (a) The admission test indicates nonrecurrent but mild variable decelerations with a slow recovery to the baseline rate at 145 bpm (black arrow). (b) Four minutes of severely prolonged deceleration with a nadir of <60 bpm, followed by a slow return to baseline (black arrow), appears after the spontaneous rupture of membranes at the second stage of labor. (c, d) Recurrent severe variable decelerations persist, becoming deeper and wider with baseline elevation during the following hour, followed by operative vaginal delivery. (The blue line for fetal heart rate tracing is obtained from a spiral electrode placed on the fetal scalp.)

**Figure 3:**
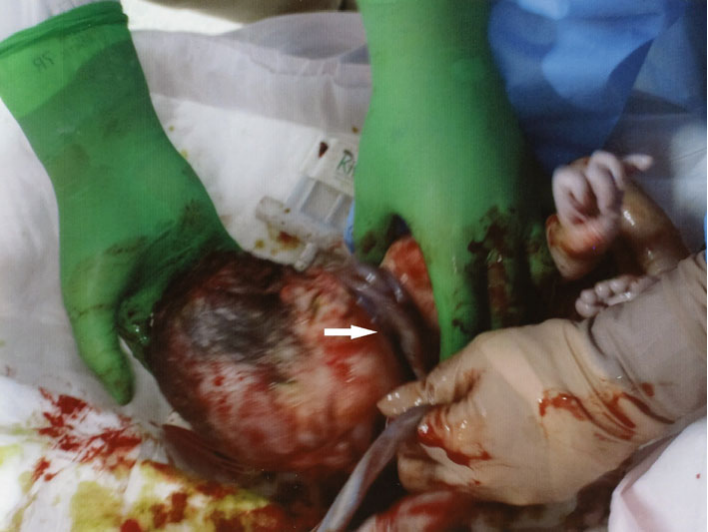
Newborn at birth with septuple tight loops of nuchal cord (white arrow).

The patient was admitted to our labor and delivery unit for spontaneous labor at 38 3/7 weeks of gestation. Her cervix was dilated by 4 cm on admission, and cardiotocography exhibited occasional mild variable decelerations with slow recovery to the baseline rate (Figure 2a). No other remarkable signs indicated fetal hypoxia, and observations were continued during the following 4 h of the first stage of labor while preparing for a cesarean section. However, 4 min of severe prolonged deceleration with a nadir FHR of <60 bpm appeared after the spontaneous rupture of the membranes along with a fully opened cervix (Figure 2b). Although this was remediated by attempting in utero resuscitative maneuvers (intravenous fluid and position change), recurrent severe variable decelerations persisted with baseline elevation during the following hour (Figure 2c). Subsequently, the patient underwent operative vaginal birth via three attempts at vacuum extraction (Figure 2d). The newborn was a male weighing 2455 g (10.5 percentile) with a septuple and tight loop of the NC (Figure 3). The umbilical artery pH was 7.31, the partial pressure of oxygen was 23.4 mmHg, and the base excess value was −6.4 mmol/L. The Apgar scores were 8 and 9 at 1 and 5 min after birth, respectively. The cord was 112 cm long with one vein and two arteries. The patient and her offspring were discharged on Postpartum Day 6 (Day 6 of life) without any notable complications. At the 1-month follow-up after birth, the infant exhibited normal growth and developmental progress. He underwent further follow-up until 4 months of age due to a systolic murmur at the left upper sternal border. Echocardiography revealed an interatrial shunt, raising concern for an atrial septum defect; however, follow-up confirmed a patent foramen ovale with spontaneous resolution.

## 3. Discussion

The present case demonstrated a distinct evolution of the FHR pattern during labor associated with umbilical cord pathology of a long cord with tight septuple NC loops. This was characterized by intermittent mild variable decelerations with the “atypical” feature (i.e., slow return to baseline) on admission, followed by progressive FHR deterioration before the operative delivery. A retrospective study of 1069 consecutive cases of severe cerebral palsy (CP) indicated that approximately one-fifth of the cases were caused by umbilical cord abnormalities [11]. Hasegawa et al. reported that severe CP cases with coexisting antenatal cord abnormalities were characterized by abnormal FHR patterns before or during the first stage of labor [12]. In the present case, vacuum-assisted delivery was performed in response to the subsequent deterioration of the FHR pattern during the second stage of labor. Although the last portion of the tracings is not severe enough to be deemed pathological (i.e., Category II in the three-tier classification or Level 3 in the five-tier classification), treatments should be directed at the underlying cause, along with in utero resuscitative maneuvers in Category II tracings with tachycardia [13]. Furthermore, the recurrent decelerations that became deeper and wider, combined with baseline elevation, represented a certain part of Hon's FHR pattern [14] and could be a risk factor for fetal acidemia and subsequent brain injury. Although the FHR decelerations with the “atypical” feature, described by Krebs in 1983 (i.e., slow return to the baseline rate, loss of variability during deceleration, or loss of primary or secondary rise in baseline rate) [15], do not indicate the need for immediate intervention [16], physicians should be aware of this umbilical cord pathology, considering the subsequent risk of FHR pattern evolution observed in the present case [17]. In addition, NC is well detectable with ultrasound at all gestational ages, with a sensitivity of 80.5% and a specificity of 86.6% [1]. The antenatal diagnosis of NC is typically based on the observation of the umbilical cord encircling at least 75% of the fetal neck in longitudinal and transverse views, and the color Doppler imaging and Doppler flow velocimetry are helpful for a more accurate diagnosis [18]. However, it may be challenging during active labor in an urgent situation. Hence, prenatal detection of umbilical cord pathology may be beneficial for the early recognition of potential fetal compromise and prompt decision-making regarding operative intervention before developing pathological FHR patterns.

The current case may have additional implications for the peripartum management of multiple NC loops. Hence, it is desirable to offer the patient an option for planned delivery even in preferable fetal well-being, considering several reports of fetal demise associated with extreme NC loops [19–21]. Furthermore, live births of six or more loops of cord entanglement may be obtained by cesarean section for abnormal FHR [22]. However, further investigations focusing on specific risk groups, including the number of entanglements, are warranted to provide an appropriate plan for individual parturients without unnecessary anxiety or intervention.

## 4. Conclusion

In summary, the present case exhibited a distinctive FHR pattern evolution, suggestive of fetal hypoxemia during labor, which is associated with multiple NC loops and a long cord. Prenatal detection of umbilical cord pathology, such as multiple entanglements, may be beneficial for early recognition of potential fetal compromise and prompt decision-making for operative intervention. However, further evidence of appropriate counseling and perinatal management for individual risk groups which focus on the number of NC loops is desirable.

## Data Availability

No underlying data was collected or produced in this study.
